# Bioactive Molecules: Structures, Functions, and Potential Uses for Cancer Prevention and Targeted Therapies

**DOI:** 10.3390/biom12091221

**Published:** 2022-09-02

**Authors:** Qingping Dou

**Affiliations:** Departments of Oncology, Pharmacology and Pathology School of Medicine, Barbara Ann Karmanos Cancer Institute, Wayne State University, Detroit, MI 48201-2013, USA; doup@karmanos.org

Cancer continues to be an increasingly pervasive and destructive disease worldwide. Although some advanced, effective cancer therapies have been developed, there are limitations in the current treatments, such as severe side effects for patients, tumor recurrence and metastasis, and the development of drug resistance. Therefore, identifying novel cancer-specific targets and developing more effective, less toxic therapeutics are of urgent clinical significance to improve the survival rate of cancer patients.

Bioactive molecules play an important role in regulating processes of cancer cell growth and development. This Special Issue of *Biomolecules*, entitled “*Bioactive Molecules: Structures, Functions, and Potential Uses for Cancer Prevention and Targeted Therapies*”, focuses on small chemical molecules from nature and biomolecules found in living organisms, as well as their synthetic family members. It is well-known that many clinically used drugs are developed from plants; many nutrient molecules from functional foods have cancer-preventive and antitumor activities, and many biomolecules (such as proteins and nucleic acids) are anticancer drug targets or have tumor-suppressing activities. All of these findings have been or could be used to develop new strategies for preventing and treating various cancers.

This Special Issue contains five (5) research articles and two (2) review articles with a focus on the reported bioactive molecules and their chemical structures, biological functions, cellular targets, signaling pathways, or mechanisms of action, as well as their potentials for cancer prevention, therapies, and management.

First, Galassi and colleagues [1] reviewed Gold(I) or Au(I)-based compounds as a promising class of bioactive molecules for the treatment of breast cancer. It is widely recognized that gold compounds are highly active and extremely potent as anticancer agents against many cancer cell lines. The presence of the metal plays an essential role in the activation of the cytotoxicity of these coordination compounds, whose activity, if restricted to the ligands alone, would be non-existent. On the other hand, gold exhibits a complex biochemistry, substantially variable depending on the chemical environments around the central metal. In this review, the scientific findings of two classes of Au(I) compounds, containing phosphane or carbene ligands, are reviewed. In addition, the recent developments in the application of Auranofin to breast cancers are discussed. Auranofin, a triethylphosphine-thiosugar compound and a drug approved by the FDA, serves as an interesting lead gold compound for understanding the activities of structurally related Au(I) compounds.

Natural xanthones are a large group of compounds from which promising anticancer properties could be further developed by chemical modifications. The study by Rech et al. [2] aimed to investigate the influence of four novel xanthone derivatives derived from a naturally occurring xanthone skeleton on the invasiveness of colon cancer cells in vitro. First, the concentrations required to inhibit the growth of three colorectal cancer cell lines to 50% (GI_50_) of all the studied compounds, as well as the natural xanthones (as a reference, including gambogic acid and α-mangostin), were measured by an MTS reduction test. Next, assays determining several aspects of the GI_25_ of xanthones on colorectal cancer cells, including cytotoxicity, migration and invasion potential, interaction with extracellular matrix and endothelial cells, as well as the expression of selected invasiveness-related genes, were performed. The results showed that these novel xanthone derivatives impair colorectal cancer proliferation, motility, and adhesion to the extracellular matrix and to endothelial cells, and also induce apoptosis and cell death. Moreover, the activities of these compounds were comparable to that of cisplatin and 5-fluorouracil (reference compounds). Further research is needed in order to develop these compounds into novel drugs for colorectal cancer treatment.

It has been reported that dienone compounds with a 1,5-diaryl-3-oxo-1,4-pentadienyl pharmacophore have tumor cell selectivity. These compounds can target the ubiquitin-proteasome system (UPS), known to be essential for the viability of tumor cells. The induction of oxidative stress, depletion of glutathione, and induction of high-molecular-weight (HMW) complexes by these compounds have also been reported. Selvaraju and colleagues [3] reported that sensitivity of acute myelocytic leukemia (AML) cells to the dienone compound VLX1570 is associated with UPS inhibition. The authors first examined the response of AML cells to VLX1570. AML cells have relatively high protein turnover rates and have also been reported to be sensitive to the depletion of reduced glutathione. The authors found that AML cells of diverse cytogenetic backgrounds are sensitive to VLX1570, with drug exposure resulting in an accumulation of ubiquitin complexes, induction of ER stress, and loss of cell viability in a dose-dependent manner. Caspase activation was observed but was not required for the loss of cell viability. Glutathione depletion was also observed but did not correlate with VLX1570 sensitivity. The formation of HMW complexes occurred at higher concentrations of VLX1570 than those required for the loss of cell viability and was not enhanced by glutathione depletion. To study the effect of VLX1570, the authors developed a zebrafish PDX model of AML and by using this model they confirmed the antigrowth activity of VLX1570 in vivo. These results demonstrate that VLX1570 induces UPS inhibition in AML cells and encourage further work in developing the compounds useful for cancer therapeutics.

Endophytic fungi are a source of compounds with unique chemical skeletons and possess interesting biological activities. Previous studies have reported that 4,6′-anhydrooxysporidinone (SSF2-2), isolated from the endophytic fungus Fusarium lateritium SSF2, has neuroprotective effects on the HT-22 hippocampal neuronal cell line. However, the anticancer effect of SSF2-2 remains unknown. Lee et al. [4] examined the viability of MCF-7 human breast cancer cells after treatment with SSF2-2 using a cell viability assay kit. They then investigated the underlying molecular mechanisms by performing Western blotting and immunocytochemistry studies. Their results demonstrated that SSF2-2 inhibited the viability of MCF-7 cells; increased the levels of cleaved caspase-9/caspase-7/poly (ADP-ribose) polymerase (PARP), and LC3B; and significantly increased the conversion of LC3-I to LC3II and LC3-positive puncta in MCF-7 cells.

Studies have demonstrated that snake venoms are a potential resource for the design of new drugs, and phospholipases A2 (PLA2), a superfamily of enzymes widely distributed in living organisms, has a broad spectrum of pharmacological activities and therapeutic potential. Van Petten de Vasconcelos Azevedo et al. [5] reported that Asp-49 BthTX-II, a PLA2-Asp-49 isolated from snake venom, inhibits tumor angiogenesis in all in vitro, ex vivo, and in vivo assays. The authors demonstrated that BthTx-II inhibits cell adhesion, proliferation, and migration of human umbilical vein endothelial cells (HUVEC), and causes a reduction in the levels of endothelial growth factor (VEGF) under conditions of in vitro angiogenesis assays. BthTx-II was also able to inhibit the sprouting angiogenic process, as shown by using the ex vivo germination assay of the aortic ring; in addition, this toxin inhibited the migration and proliferation of HUVEC under co-culture conditions with triple-negative breast cancer cells (e.g., MDA-MB-231 cells). Finally, the authors determined the in vivo tumor suppression and anti-angiogenic activities of BthTx-II by injecting MDA-MB-231 cells (plated in Matrigel) into the chorioallantoic membrane of a chicken embryo (CAM). Taken together, these results demonstrate an important antiangiogenic and antitumor role of BthTx-II that has great potential to be developed into a novel antitumor drug for cancer therapy.

The next collection is a review article of Zeng, Jiang, and Sanders [6] focusing on cellular and molecular prospects of EPLIN (Epithelial Protein Lost In Neoplasm) in cancers. EPLIN, an interesting protein molecule, which is also known as LIMA1 (LIM Domain Additionally, Actin Binding 1), was first discovered as a protein differentially expressed in normal and cancerous cell lines. Despite a slow pace in early years in understanding its biological role in cells and body systems as well, as its clinical implications, recent years have witnessed rapid progress in understanding the mechanisms of this protein in cells, diseases, and the body. It is now well known that EPLIN is key to the progression and metastasis of certain solid tumors. EPLIN has drawn more attention over the past few years with its roles expanding from cell migration and cytoskeletal dynamics, to cell cycle, gene regulation, angiogenesis, lymphangiogenesis, and lipid metabolism. This concise review article summarizes and discusses the recent progress in EPLIN, its biological processes, and implications in cancer.

The final article in this Special Issue is a report from Cho and colleagues [7] on non-coding RNAs (ncRNAs) from plasma exosomes as a new method for cervical cancer diagnosis. The authors first collected differentially expressed RNAs from a group of 12 healthy individuals (normal group) and a pretreatment group of 30 patients with cervical cancer (cancer group). They then analyzed the association between an ncRNA and mRNA network and cancer by using ingenuity pathway analysis, according to the number and correlation of mRNAs (or ncRNAs) relative to changes in the expression of primarily selected ncRNAs (or mRNAs) before and after chemoradiotherapy. They showed that the combination of miRNA, mRNAs, and snoRNAs gave clearly distinguished profiles between the normal and the cancer samples. Therefore, the authors have presented a new method for efficiently screening RNAs isolated from exosomes for cervical cancer diagnosis.

I anticipate that, taken together, these timely reports will provide inspiration for the field of antitumor drug development, cancer research, and management. Although remarkable progress has been made, the field still faces some challenges as recognized by the authors. Regardless, the future of using bioactive molecules for cancer treatment remains bright. I would like to thank all the authors—who are experts in the abovementioned research areas—for their remarkable contributions.
